# Glycogen and Glucose Metabolism Are Essential for Early Embryonic Development of the Red Flour Beetle *Tribolium castaneum*

**DOI:** 10.1371/journal.pone.0065125

**Published:** 2013-06-04

**Authors:** Amanda Fraga, Lupis Ribeiro, Mariana Lobato, Vitória Santos, José Roberto Silva, Helga Gomes, Jorge Luiz da Cunha Moraes, Jackson de Souza Menezes, Carlos Jorge Logullo de Oliveira, Eldo Campos, Rodrigo Nunes da Fonseca

**Affiliations:** 1 Laboratório Integrado de Bioquímica Hatisaburo Masuda (LIBHM), Núcleo de Pesquisas Ecológicas e Sócioambientais de Macaé (NUPEM), Universidade Federal do Rio de Janeiro (UFRJCampus Macaé), Rio de Janeiro, Brazil; 2 Programa de Pósgraduação em Produtos Bioativos e Biociências (PPGPRODBIO), Universidade Federal do Rio de Janeiro (UFRJCampus Macaé), Rio de Janeiro, Brazil; 3 Laboratório de Química e Função de Proteínas e Peptídeos and Unidade de Experimentação Animal, Universidade Estadual Norte Fluminense Darcy Ribeiro (UENF), Campos dos Goytacazes, Rio de Janeiro, Brazil; 4 Instituto Nacional de Ciência e Tecnologia em Entomologia Molecular, Rio de Janeiro, Brazil; Iowa State University, United States of America

## Abstract

Control of energy metabolism is an essential process for life. In insects, egg formation (oogenesis) and embryogenesis is dependent on stored molecules deposited by the mother or transcribed later by the zygote. In oviparous insects the egg becomes an isolated system after egg laying with all energy conversion taking place during embryogenesis. Previous studies in a few vector species showed a strong correlation of key morphogenetic events and changes in glucose metabolism. Here, we investigate glycogen and glucose metabolism in the red flour beetle *Tribolium castaneum*, an insect amenable to functional genomic studies. To examine the role of the key enzymes on glycogen and glucose regulation we cloned and analyzed the function of *glycogen synthase kinase 3 (GSK-3)* and *hexokinase (HexA)* genes during *T. castaneum* embryogenesis. Expression analysis via *in situ* hybridization shows that both genes are expressed only in the embryonic tissue, suggesting that embryonic and extra-embryonic cells display different metabolic activities. dsRNA adult female injection (parental RNAi) of both genes lead a reduction in egg laying and to embryonic lethality. Morphological analysis via DAPI stainings indicates that early development is impaired in *Tc-GSK-3* and *Tc-HexA1* RNAi embryos. Importantly, glycogen levels are upregulated after *Tc-GSK-3* RNAi and glucose levels are upregulated after *Tc-HexA1* RNAi, indicating that both genes control metabolism during embryogenesis and oogenesis, respectively. Altogether our results show that *T. castaneum* embryogenesis depends on the proper control of glucose and glycogen.

## Introduction

Energy homeostasis is an essential process for life [1]. Carbon source conversion in living tissues involves tight regulation of enzymes of the glycolytic pathway. During oogenesis, the insect mother deposits lipids, proteins, carbohydrates and mRNAs which are essential for posterior embryonic development. Particularly in oviparous species the insect egg must contain all nutrients required for embryonic development being a closed and isolated system from the environment [2]. Classical insect studies have investigated metabolic activity in several organs during adulthood and oogenesis [3], [4], [5], [6], [7], but only recently metabolic activity during embryogenesis was analyzed in a few arthropod species [8], [9].

**Figure 1:**
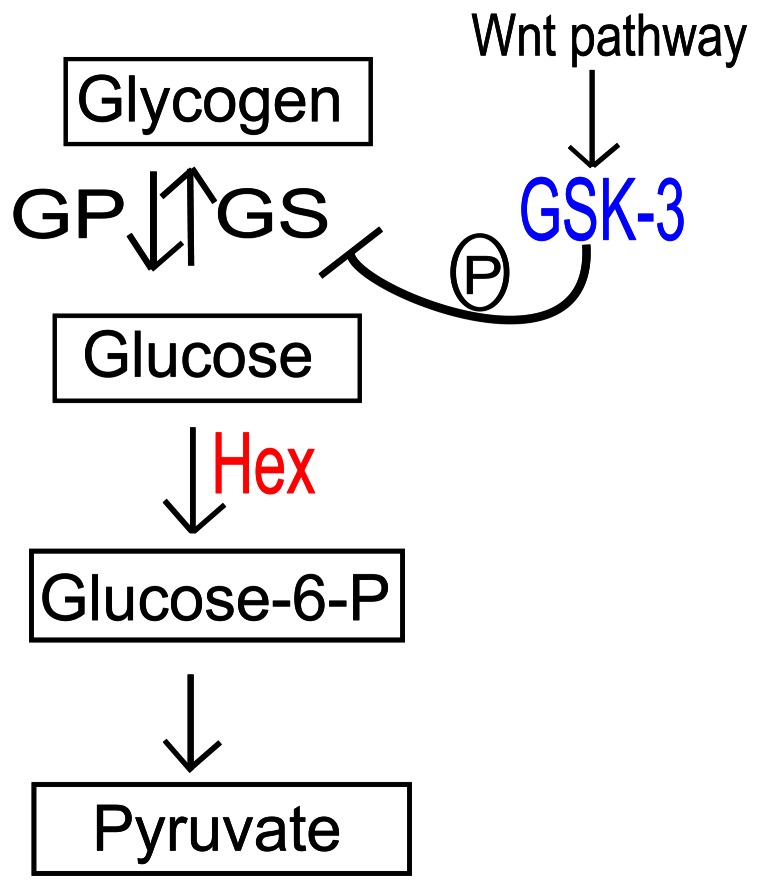
Simplified view of the metabolic pathways investigated in this study. Glycogen Synthase Kinase-3 (GSK-3-blue) is not only involved in glycogen synthase (GS) regulation, but also acts as a downstream component of the Wnt signaling pathway (*e.g.* [60]). Glycogen Phosphorylase (GP) breaks up glycogen into glucose subunits. Hexokinase (Hex-red) is involved in producing Glucose-6-P from glucose, acting as an important enzyme of the glycolytic pathway. Other Glucose-6-P possible roles in different metabolic pathways are omitted for simplicity.

A simplified model of energy utilization of glycogen mobilization into the glycolytic pathway (Figure 1) shows that glycogen storage is mobilized into the glycolytic pathway due to the action of Glycogen Phosphorylase (GP), while its synthesis is due to Glycogen Synthase (GS), whose activity is regulated by glycogen synthase kinase-3 (GSK-3) (reviewed in [10]). GSK-3 is not only involved in metabolic reactions but also acts as an essential kinase of the Wnt pathway [11], [12], which plays an essential role during embryogenesis. Recently, GSK-3 was also shown to be involved in the integration of the Wnt and BMP/Smad1 pathways [13], [14], [15]. Thus, GSK-3 is an important “hub” linking important signaling pathways during embryogenesis to metabolic reactions.

Glycogen break down generates glucose, which enters in the glycolytic pathway being converted into pyruvate (Figure 1). This process leads to ATP generation. Important enzymes for this process such as Hexokinase (Hex or HK) have been characterized in several organisms [16], [17], [18], [19], [20]. Hexokinase (ATP: hexose-6-phosphotransferase, E.C. 2.7.1.1; Hex) catalyzes the first step in the oxidative metabolism of hexoses via glycolysis. Four distinct hexokinase isozymes are reported for mammalian tissues and are named as types I–IV (also called types 1–4 or A–D). Structurally, Hex I–III are 100 kDa proteins thought to have evolved by duplication and fusion of a gene encoding an ancestral 50 kDa hexokinase [16], [17], [21]. In insects, classical studies have isolated four Hex isozymes from different tissues of the fruit fly *Drosophila melanogaster* [22], [23]. After *D. melanogaster* genome sequencing four genes encoding Hex proteins were identified [24], [25], [26]. Only recently *Hex* from the shrimp *Litopenaeus vannamei* was cloned and shown to be regulated by hypoxia as its mammal homolog [27].

Previous studies have analyzed metabolic regulation during embryogenesis in blood sucking arthropod species like the tick *Riphicephalus (Boophilus) microplus* [8] and the mosquito *Aedes aegypti* [9]. These studies have revealed important morphogenetic events which are associated with changes in the embryonic metabolic regulation, *e.g.,* germ band retraction is correlated with an increase in glycolysis during mosquito embryogenesis [9].

Here, we have investigated the metabolic regulation in the red flour beetle *Tribolium castaneum,* which has emerged in the past few years as an excellent model for studies of embryogenesis and evolution of signaling pathways (reviewed in [28], [29]). This beetle had its genome sequenced [30], is amenable to functional studies like RNA interference (RNAi) [31], [32], and mutant and enhancer trap lines have been developed [33]. *T. castaneum* feeds on whole grain flour during all its motile stages. This stands in great contrast to the species previously analyzed like mosquitos and ticks [8], [9], which do not feed continually and ingest huge amounts of blood in each occasional feeding session.

In this study we characterized *T. castaneum* metabolic status during early embryogenesis. Our results show that glucose and glycogen regulation are important for early *T. castaneum* embryonic patterning. Expression, activity and functional analysis of *Tc-HexA1* and *Tc-GSK-3* suggest important roles of these enzymes during oogenesis and embryogenesis.

## Methods

### Tribolium Castaneum Strains

San Bernardino beetles are reared at 30°C in wheat flour supplemented with 5% dried yeast. The beetles were maintained inside plastic boxes of approximately 15×15 cm with humidity between 40–80% as previously described [29].

### Primer Design and Expression Analysis

Orthologs of Glycogen Synthase Kinase-3 (GSK-3) (GSK-3) and Hexokinase (Hex) were identified in the *Tribolium castaneum* genome [30] by BLAST. Hex and GSK-3 protein sequences were aligned with ClustalW (http://www.ebi.ac.uk/clustalw) to several family members known in other vertebrate and invertebrate species. Parts of the alignment where most sequences had gaps were not taken into account for phylogenetic analysis by creating a mask in Seaview. The most informative amino acid substitution model was calculated with Prottest [34]. Maximum likelihood phylogenies were generated with PhyML [35]. Trees were edited in MEGA5.05 [36]. Primers for *Tribolium castaneum HexokinaseA1* (Glean_00319) and *GSK-3* were designed with Primer3 containing the following sequences: *Tc-HexA1-5′*-ggccgcgggACACGAGGTTTTACCGTTGG, *Tc-HexA1-3′*- cccggggcGAGAAATGCATTCGCAGACA, Tc-*GSK-3*-5*′* ggccgcgggACCAAAGTTATCGGCAATGG and Tc-*GSK-3*-3*′*-cccggggcGCCACTAACTCGATCGCTTC. The sequences in lowercase are adaptor sequences which enable the primers to be used as future templates for synthesis of anti-sense RNA probe or of double-strand RNA (dsRNA) [37]. The amplicon size for these primer pairs are 721bp for Hexokinase A1 (*Tc-HexA1*) and 778 bp for *Tc-GSK-3*. For *Tc-HexA1* the dsRNA construct cover the 471-1192 nucleotide positions of a predicted transcript of 1434 bp. For *Tc-GSK-3* the dsRNA construct cover the 406-1184 nucleotide positions of a predicted transcript of 1485 bp. Unrelated dsRNA (LacZ) was used as a negative control during injections. Another construct was injected for each gene as a control for off-target effects lead to identical knockdown phenotypes. BLAST searches at NCBI did not show any significant similarity against other genes of the *Tribolium* genome [30] discarding off-targets effects.

### *in situ* Hybridization and RNAi

Double-stranded RNA (dsRNA) was synthesized using T7 MEGAScript (Ambion), purified and injected in adult females as previously described [32]. *In situ* hybridization was performed using digoxygenin labeled RNA probes, and revealed with alkaline phosphatase chromogenic substrate BM Purple (Roche). The one-color *in situ* protocol for *Tribolium* was done as described by [38] followed by nuclear DAPI staining (4′,6-diamidino-2-phenylindole) before documentation. A sense probe of each gene was included during *in situ* hybridization experiments and did not show any specific staining.

### Real-time PCR: Quantitative Real-time PCR

Total RNA was isolated from 100 mg of eggs collected from specific development stages using Trizol® (Invitrogen) according to the manufacturer’s instructions. First strand complementary DNA (cDNA) was synthesized using Superscript III reverse transcriptase (Invitrogen) and real time PCR analysis using SYBR green based detection was performed. Reactions were carried out in triplicate, and melting curves were examined to ensure single products. Results were quantified using the “delta-delta Ct” method and normalized to *rps3* transcript levels and to control genotypes [39]. Data shown are averages and standard deviations from at least three independent experiments.

### Determination of Glucose and Glycogen Content

At least 10 mg of eggs were collected and submitted to a 3 minute bleach treatment to remove flour and chorion. Then, eggs were dried on a filter paper (Whatman) and glucose content enzymatically quantified by glucose oxidase (glucox® enzymatic Kit for glucose dosage; Doles, inc.). After 30 min of incubation at 37°C, the samples were read at 510 nm in a Shimadzu spectrophotometer, according to the manufacturer’s instructions. Glucose content was determined using a standard curve submitted to the same conditions [9]. For glycogen determination, eggs were prepared as described above and homogenized in a buffer containing 200 mM sodium acetate, pH 4.8. The homogenate was incubated with 1 unit of a-amyloglucosidase (Sigma Chemicals) for 4 hours at 40°C. The newly generated glucose was enzymatically determined by glucose oxidase as previously described. Free glucose was subtracted from samples without α-amyloglucosidase. Glycogen content was determined using a standard curve submitted to the same conditions [9].

### Hexokinase Enzymatic Activity

*Cytoplasm isolation:* The cell fractionation procedure used required large amounts of fresh eggs (at least 0,056 g respective to 250 eggs) to obtain the cytoplasm fraction. Eggs were homogenized in 1 mL of a buffer containing 0.5 M sucrose, 50 mM Tris-HCl pH 7.4, 100 µM leupeptin, 100 nM pepstatin and 20 mM MgCl_2_. The homogenate was centrifuged at 200 g for 2 min. The supernatant was carefully removed and centrifuged at 100.000 *g* for 1 hour for obtain the cytoplasmic fraction in the supernatant. *Hexokinase (Hex) cytoplasmic activity assay*: The samples were assayed in 20 mM Tris-HCl pH 7.4 containing 6 mM MgCl_2_, 1 mM ATP, 0.5 mM NAD^+^ and 2 mM glucose. Hex catalytic activity was measured by adding Leuconostoc mesenteroides glucose 6-phosphate dehydrogenase (Sigma-Aldrich Chemicals) (Worthington Code: ZF or ZFL) dissolved at a concentration of 1 UI/mL in the above Tris-MgCl_2_ buffer [40]. The production of β-NADH was monitored at 340 nm in a Shimadzu spectrophotometer using a molar extinction coefficient of 6.22 M-1 [41].

### Comparison of Glucose Content in Control (*LacZ* RNAi) and *Tc-HexA1* RNAi Ovaries

Fifty females were injected either with *Tc-HexA1* or *LacZ* dsRNA at 1 µg/µl. Two days after injection, males were added and egg number scored every 48 hours in both groups. Since after *Tc-HexA1* dsRNA injection oviposition is almost completely abolished, 10 ovaries were dissected from control and *Tc-HexA1* dsRNA females five days after dsRNA injection. These ovaries either had their morphology analyzed by nuclear DAPI stainings or were submitted to glucose measurement as described above. Three independent biological replicates were performed. Glucose values were normalized in relation to the protein amount (Bradford method).

### Comparison of Glycogen Content in Control (*LacZ* RNAi) and *Tc-GSK-3* Embryos

Fifty females were injected either with *Tc-GSK-3* or LacZ dsRNA at 50 ng/µl. Two days after female injection, males were added and eggs collected after 48 hours (0–48 hours) for both control and *Tc-GSK-3* RNAi eggs. Both groups of eggs were let for an additional 24 hour period (48–72 hours). Three independent biological replicates were performed. Glycogen content was measured as described in [9] and the protein amount (Bradford method) was used to normalize each sample.

## Results

The knowledge of glycogen and glucose energy control during embryonic development is quite scarce and restricted to arthropod vectors [8], [9]. Since all previous analysis were performed on blood feeders that have restricted access to food sources and have to cope with heme toxicity we sought to investigate how a beetle with unlimited access to starch would store its energy supplies and transfer them to the eggs.

### Glucose Content is Low during Early Stages and High during Late Embryogenesis

**Figure 2:**
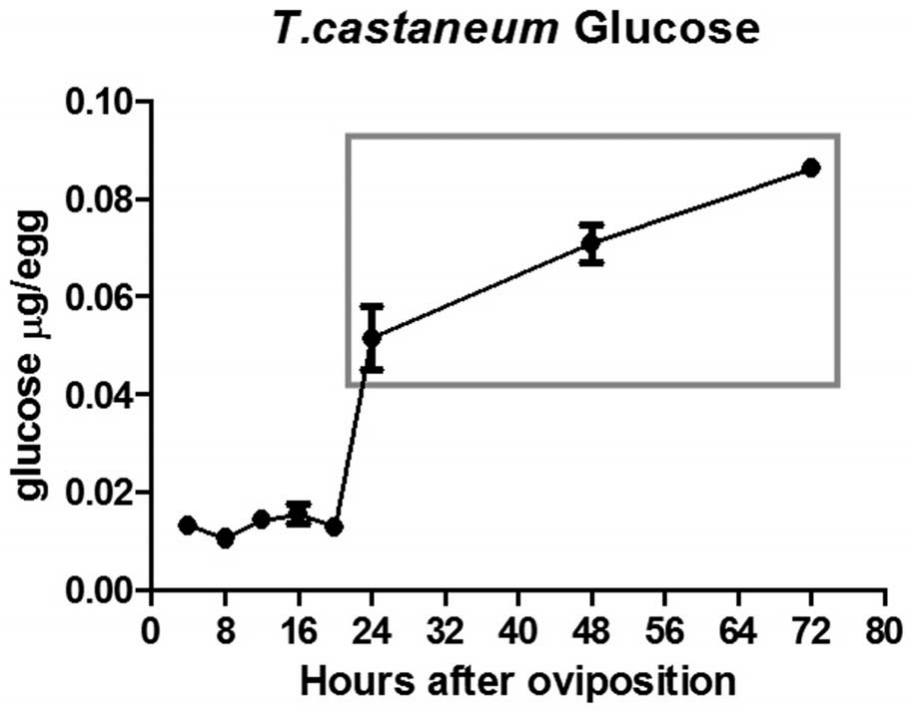
Analysis of glucose content during *Tribolium castaneum* embryogenesis. Glucose content is low during the first 20 hours of embryonic development, and increases from 20 hours on towards maximal levels until larvae hatching (about 96 hours after egg laying). Grey box highlights the region with high glucose content.

Since glucose is a major energy source for living tissues we measured the glucose content during *Tribolium castaneum* embryogenesis with particular emphasis on the first 72 hours of development when most cell proliferation and morphogenesis takes place (Figure 2). Glucose levels remain low during the first 20 hours of development. Later, between 20–24 hours a large increase of glucose is observed, which is further reinforced in the next two days of development (24–48 and 48–72 hours). Importantly, after this increase levels are 60% higher than the 0–4 hours of embryonic development. Thus, glucose content changes during *T. castaneum* embryonic development.

### Tribolium Castaneum Genome Contains Two HexokinaseA (HexA) Genes

Since glucose levels are dependent on the action of Hexokinase (Hex), an enzyme that converts glucose into glucose-6-P, we searched in the *T. castaneum* genome [30] for putative Hex sequences. As previously described [24], [26], four *Hex* genes exist in the *D. melanogaster* genome, while all other insect genomes analyzed so far seem to contain only one *Hex* gene. This unique *Hex* gene observed in other insect genomes is more similar in sequence to *HexA* from *D. melanogaster* than to other Hex genes.

**Figure 3:**
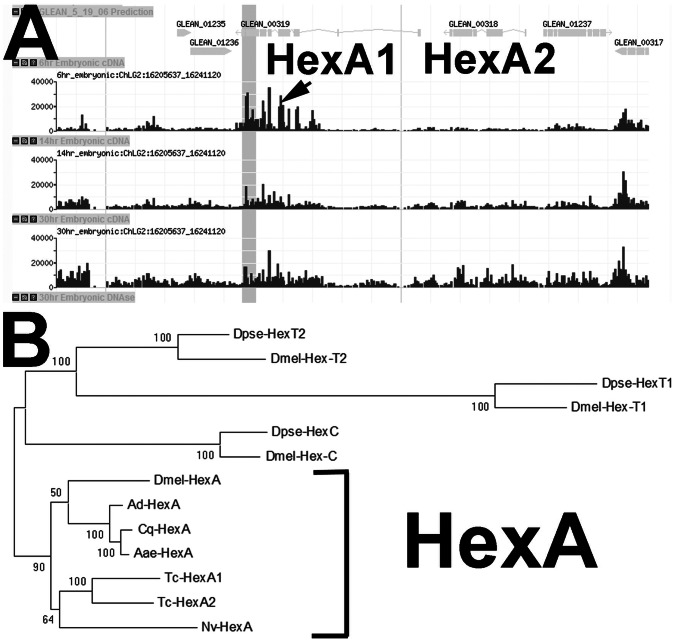
*Hexokinase (Hex)* locus structure in *Tribolium* and *Hex* gene evolution in insects. (A) Snapshot of the Beetlebase [42], [43] showing relative expression of *Tc-HexA1* (Tc-Glean00319) and *Tc-HexA2* (Tc-Glean00318) at 6 hours, 14 hours and 30 hours of embryonic cDNA libraries. Note that *Tc-HexA1* is expressed at early stages while *Tc-HexA2* seems to be upregulated only at later stages. (B) Phylogenetic analysis using maximum likelihood method. Amino acid substitution model: WAG+G. In Drosophillids four *Hex* genes exist (*HexC, HexT1, HexT2 and HexA),* while in most other insects only one *Hex* gene exists. Bootstrap values (1,000 replicates) are indicated as percentages. *Aae - Aedes aegypti*; *Ad - Anopheles darling*; *Am - Apis mellifera; Cq - Culex quinquefasciatus; Dmel - Drosophila melanogaster; Dpse - Drosophila pseudoobscura; Nv - Nasonia vitripennis; Tc - Tribolium castaneum*. Accession numbers for the NCBI are available upon request.

Interestingly, *T. castaneum* contains two *Hex* genes arranged in tandem in the genome suggesting a recent duplication event (Figure 3A). Both genes *Tc-HexA1* (Glean_00319) and *Tc-HexA2* (Glean_00318) encode proteins more similar in amino acid sequence to HexA from other insects (Figure 3B). Both genes display high identity to each other (over 80% at amino acid level). Closer inspection of the *Tc-HexA* genes using expression arrays of the BeetleBase [42], [43] revealed interesting features of this locus, which contains both genes *Tc-HexA1* and *Tc-HexA2*. *Tc-HexA1* is highly expressed during early embryogenesis at six hours of embryonic development, while *Tc-HexA2* seems to be upregulated only at later embryonic stages (Figure 3A-30 hours). Since our major interest was to investigate the metabolism during early embryogenesis, we analyzed the expression and function of *Tc-HexA1,* the early expressing hexokinase from *T. castaneum.*

### Hexokinase Expression and Activity Suggests an Early Role during *Tribolium castaneum* Embryogenesis

**Figure 4:**
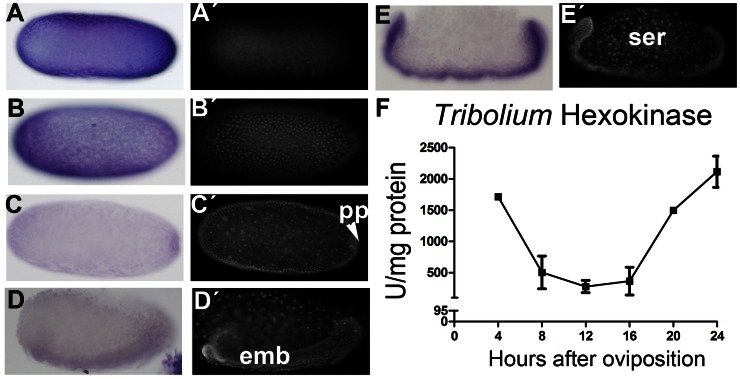
*In situ* hybridization of *Tc-HexA1* and Hexokinase activity during the first 24 hours of beetle embryogenesis. (A–E) *In situ* hybridization and respective nuclear DAPI stainings (A’–E’). In all panels head is to the left and dorsal side up. (F) Hex activity during the first 24 hours of embryonic development. (A,A’) Eggs during the first four hours after egg lay (AEL), when rapid cleavages occur display ubiquitous *Tc-HexA1* mRNA. (B,B’) Eggs between four and eight hours (4–8 hours) also show ubiquitous *Tc-HexA1* expression. (C,C’) During gastrulation between 8–12 hours *Tc-HexA1* expression largely decreases, remaining low between 12–16 hours in D,D’. (E,E’) During germ band elongation (16–20 hours) *Tc-HexA1* expression is upregulated and occurs only in the embryonic region (emb in D), being absent in the serosa (ser). (F) Specific Hexokinase activity (U/mg protein). High activity is detected in egg extracts from 0–4 hours and after 16 hours, which correlates to *Tc-HexA1* mRNA expression pattern. pp - posterior pit, emb - embryonic tissue, ser - serosa.

Since *Tc-HexA1* appears to be expressed during early hours of embryogenesis we investigated its mRNA localization by *in situ* hybridization. *Tc-HexA1* is detected ubiquitously during the first four hours of embryonic development probably due to maternal mRNA deposition (Figure 4A). At that stage only a few nuclei can be observed by nuclear DAPI stainings (Figure 4A *’*). During the next four hours of embryonic development extensive cell division takes place and *Tc-HexA1* expression is still observed (Figure 4B, B’). Soon after, *Tc-HexA1* expression starts to diminish and the lowest levels are observed shortly before gastrulation (8–12 hours), when the posterior pit (pp) can be observed (Figure 4C,C’). During gastrulation and beginning of germ band extension mRNA levels remain low (Figure 4D) and the embryonic (emb) cells at the ventral side can be distinguished from the serosa cells, the latter with large nuclei (Figure 4D’). During germ band extension (16–20 hours) *Tc-HexA1* expression is upregulated and identified only at the embryonic region; expression in the polyploid serosa (ser) cells is absent (Figure 4E, E’). Taken together, the spatial analysis of *Tc-HexA1* expression suggest a temporal control at early stages (0–12 hours) and a spatial control shortly after (16–20 hours) with embryonic cells expressing this enzyme and extra-embryonic cells lacking it (Figure 4D,E).

These results stimulated the investigation of Hexokinase (Hex) activity during similar stages of embryogenesis. Thus, we isolated the cytoplasmic fraction of eggs in intervals of four hours of embryonic development in order to measure specific Hex activity. High levels of Hex activity are observed in the first four hours of development (0–4 hours - Figure 4F). A decrease to about one-third of the initial activity is observed in the 4–8 hours of development and this low level is maintained for the next 8 hours, 8–12 and 12–16 hours of embryonic development. Between 16–20 hours an upregulation of Tc-Hex activity is observed, which is further increased in the next four hours (20–24 hours - Figure 4F). This activity remains high until the end of embryogenesis (data not shown). Comparison of Hex activity (Figure 4F) and *Tc-HexA1 in situ* expression (Figure 4A–E) suggest a correlation between transcriptional level and enzymatic activity during early embryogenesis.

### pRNAi against *Tc-HexA1* Shows a Major Requirement of this Enzyme for Oogenesis and Embryogenesis

To investigate if *Tc-HexA* is important for embryonic development, parental RNAi (pRNAi) was performed as previously described for several other genes in this species [44], [45], [46], [47].

**Figure 5:**
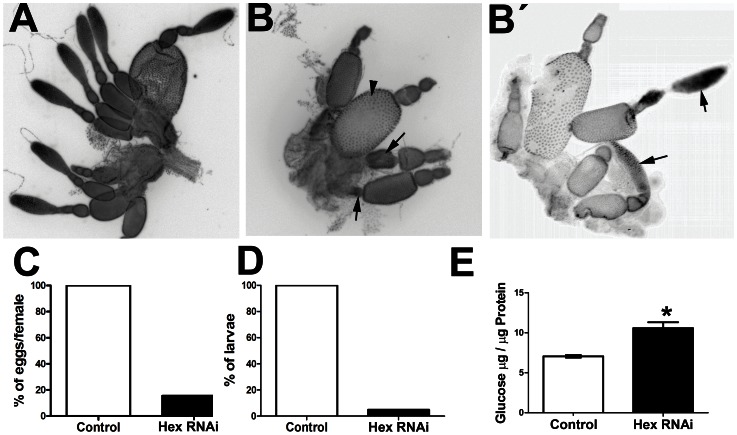
*Tc-HexA* RNAi affects oogenesis, glucose content, and reduces egg lay. (A,B) Ovary morphology in (A) control ovaries (injected with LacZ dsRNA) and (B,B’) After *Tc-HexA1* dsRNA injection. (B)*Tc-HexA1* dsRNA ovarioles are less numerous and display many oocytes undergoing apparent degeneration (black arrows) when compared to the control ovaries. Mature oocytes can be eventually identified in *Tc-HexA1* dsRNA ovaries (arrowhead). Nurse cells of the *Tc-HexA1* dsRNA ovarioles also appear reduced when compared to the control, although the germarium in some ovarioles seem not to be affected like in B’. (B’) Arrowheads highlights the germarium in *Tc-HexA1* dsRNA ovaries, which appears similar to the control in some ovarioles. (C) *Tc-HexA1* dsRNA injection largely reduces oviposition when compared to the WT. (D) Analysis of larvae hatching after *Tc-HexA1* RNAi when compared to the control. Less than 10% of the laid eggs hatch, indicating an essential role of *Tc-HexA1* during embryonic development. (E) Analysis of glucose content in ovaries injected with *Tc-HexA1* dsRNA and the control (LacZ dsRNA). Asterisk indicates that the difference between the two groups is statistically significant (p<0,05).

In all experiments, we injected the unrelated dsRNA LacZ as a negative control in a separate batch of females. These LacZ dsRNA females laid the normal amount of eggs, which hatched as larvae, indicating that injection of unrelated dsRNA had no effect on *T. castaneum* development. We then analyzed several parameters related to fecundity in the females injected with *Tc-HexA1* dsRNA. First, egg laying of the *Tc-HexA1* dsRNA injected females was drastically reduced to 10% of the control (Figure 5C). Second, among the few *Tc-HexA1* RNAi laid eggs, only 5% of them hatched as larvae, indicating a strong requirement of this gene for embryonic development (Figure 5D). This extreme reduction in egg laying prevented the analysis of Hex activity or glucose content in RNAi embryos. We tried to circumvent this problem by analyzing glucose content in ovaries of *Tc-HexA1* dsRNA and LacZ dsRNA (control) injected females. Interestingly, glucose content was higher in *Tc-HexA1* dsRNA ovaries when compared to the control (Figure 5E). This reduction in egg laying stimulated us to compare ovary morphology in control and *Tc-HexA1* dsRNA injected females. Morphological analysis via nuclear DAPI stainings of the ovarioles of control and *Tc-HexA1* dsRNA injected females showed clear differences (Figure 5A,B). *T. castaneum* control ovaries display several tube-like projections, the ovarioles *e.g.* [37], which contains oocytes in different stages of maturation. In control ovaries, larger eggs are present in the distal part of the ovariole. *Tc-Hex* RNAi ovarioles showed distinct features. First, the ovariole number is reduced (Figure 5B and data not shown). Second, degenerated oocytes can be observed at the distal part (Figure 5B - black arrows), although some mature oocytes surrounded by follicle cells can also be observed (Figure 5B - arrowhead). Third, the germarium appears to be diminished in some *Tc-HexA1* RNAi ovarioles (Figure 5B), while others appear similar to control ovaries (Figure 5B’-arrows). This abnormal ovary morphology is probably related to the large reduction in oviposition when compared to the control (Figure 5C). Finally, the few eggs observed after *Tc-HexA1* RNAi stopped embryonic development before cellularization (8 hours of development - data not shown). To sum up, the analysis of *Tc-HexA1* role during oogenesis and embryogenesis supports an essential role of *Tc-HexA1* and glucose metabolism during these processes. Since glucose can be generated via glycogen degradation we sought to investigate how glycogen is regulated during *T. castaneum* embryogenesis.

### Glycogen is Degraded in Two Phases throughout Embryogenesis

**Figure 6:**
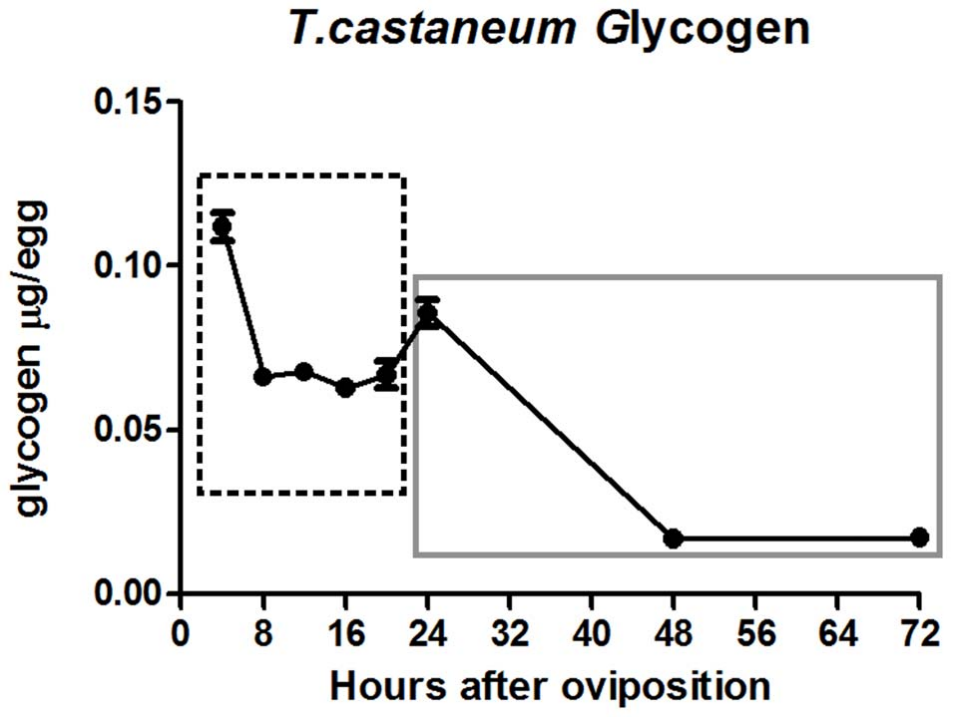
Glycogen content decreases in two phases during *T. castaneum* embryogenesis. High glycogen content is detected at the first four hours of embryogenesis and decreases between 4 and 8 hours of embryonic development (dashed box). Glycogen level is maintained or slightly increased between 8–12, 12–16, 16–20 and 20–24 hours. During the next 24 hours glycogen content largely decreases (grey box) and remains low until 72 hours.

Changes in glucose levels (Figure 2), in Hex activity (Figure 4F) and *Tc-HexA1* expression (Figure 4A–E) suggest a tight control of beetle embryonic metabolism. Particularly important is the possibility that glucose upregulation observed in the second embryonic phase, after 24 hours, could be generated by glycogen conversion into glucose. To investigate this hypothesis we measured glycogen content. High glycogen content was observed in the first four hours of embryogenesis (0–4 hours) when compared to later stages (Figure 6). During the next five time-points of four hours (4–8, 8–12, 12–16, 16–20 and 20–24 hours) there is no significant change on glycogen levels with the exception of a small increase between 20–24 hours. Later on, a huge decrease in glycogen level occurs between 24–48 hours, leading to a basal level, whichis maintained in the next day of development (48–72 hours). To sum up, glycogen is degraded in two phases throughout embryogenesis and it is important to investigate the mechanism responsible for this regulation.

### *Tc-GSK-3* is Maternally Deposited and Expressed Only in the Embryonic Rudiment

**Figure 7:**
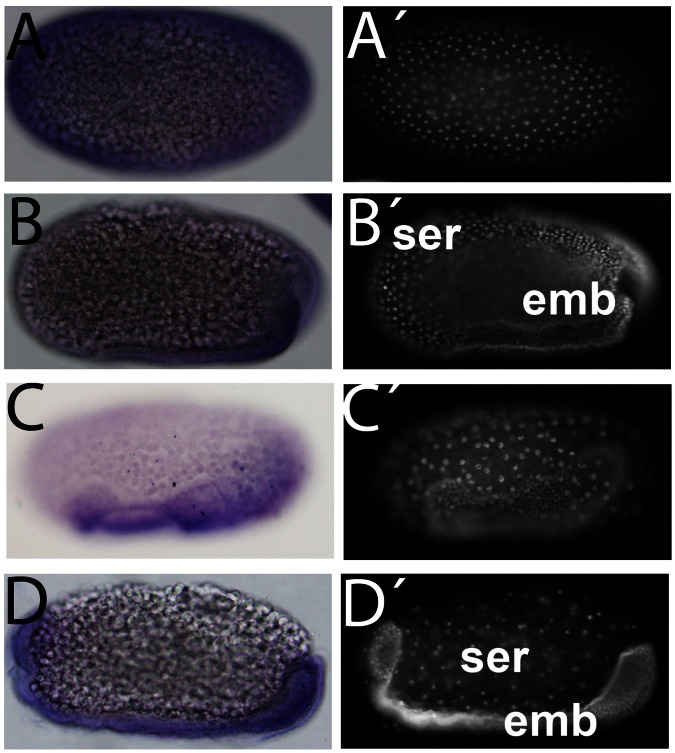
*Tc-GSK-3* is expressed in the embryonic tissue throughout embryogenesis. (A–D) *Tc-GSK-3* expression and respective nuclear DAPI stainings (A’–D’). (A,A’) In the first four hours after egg laying (AEL), *Tc-GSK-3* is ubiquitously expressed. Note the few nuclei at the periphery. In the next four hours (4–8 hours) a similar ubiquitous expression pattern is observed (data not shown). (B–B’) Between 8–12 hours AEL *Tc-GSK-3* expression is mainly concentrated at the embryonic cells (emb) and not in the extraembryonic polyploid serosal (ser) cells. (C–C’) This pattern of strong expression of *Tc-GSK-3* in the embryonic cells remains between 12–16 hours AEL, when serosal cells surround the embryonic ones. (D–D’) Between 16–20 hours AEL *Tc-GSK-3* expression is still largely confined to the embryo (emb), which is undergoing germ band elongation. At 20–24 hours AEL a similar expression profile is observed (data not shown).

One of the key enzymes involved in glycogen regulation and degradation is the glycogen synthase kinase (GSK-3), which phosphorylates Glycogen Synthase (GS) responsible for glycogen synthesis. Phosphorylation of GS by GSK-3 decreases its activity. In addition to its metabolic role, GSK-3 also acts as a key downstream component of the Wnt pathway (reviewed in [48]). To investigate the role of GSK-3 during *T. castaneum* embryogenesis we performed *in situ* hybridization using embryos collected every four hours as previously described for *Tc-HexA1*. First, *Tc-GSK-3* seems to be maternally provided since its mRNA is detected along the whole egg during the first four hours of development (Figure 7A,A’). In the interval between 4 and 8 hours and between 8–12 hours, *Tc-GSK-3* is detected only at the germ rudiment, a ventral-posterior part of the egg, constituted by embryo and the amnion (Figure 7B,B’-emb, data not shown), and not in the extra-embryonic serosa (Figure 7B, ser, see also [49]).

When the serosal cells start to cover the embryonic region during 12–16 hours and during the beginning of germ band elongation (16–20 hours), *Tc-GSK-3* expression is detected only at the embryonic tissue with ubiquitous mRNA expression in the embryo (Figure 7C,C’, D, D’ - emb). Again, *Tc-GSK-3* is not expressed in the serosa (Figure 7C,D - ser). These results suggest that metabolic regulation in the embryo is different from the extra-embryonic cells (serosa).

### pRNAi Analysis Shows an Essential Role of *Tc-gsk-3* during Early Embryogenesis

**Figure 8:**
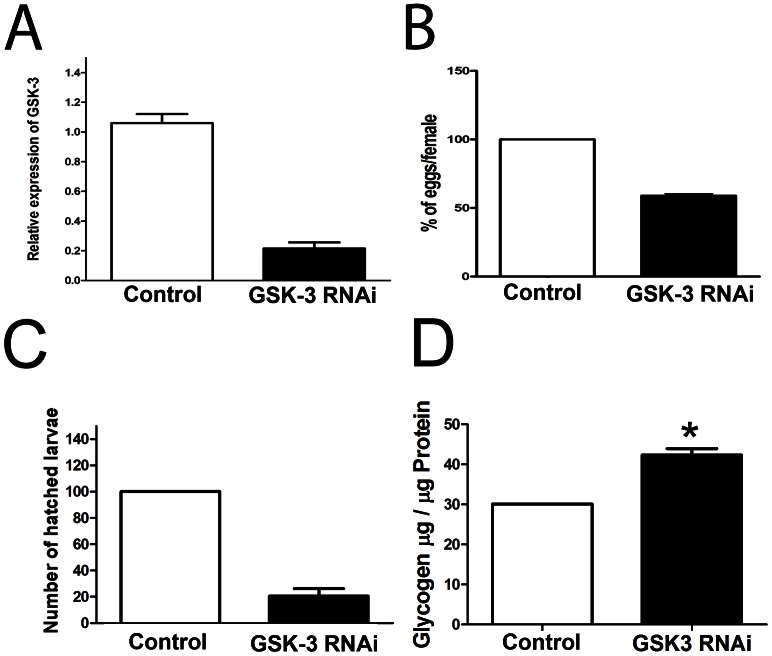
*Tc-GSK-3* knockdown affects *T. castaneum* egg laying, larvae hatching and glycogen content. (A) *Tc-GSK-3* expression decreases after *Tc-GSK-3* dsRNA injection. (B) Number of laid eggs diminishes 50% after *Tc-GSK-3* RNAi when compared to the control. (C) Number of hatching larvae decreases after *Tc-GSK-3* RNAi to about 20% of the control. (D) Glycogen content increases in *Tc-GSK-3* RNAi eggs when compared to the control (*LacZ* RNAi eggs). Asterisk indicates that the difference between the two groups is statistically significant (p<0,05).

Recently, during the writing of this manuscript, Bucher and co-authors have published a throughout description of *Tc-GSK-3/shaggy* RNAi phenotype [44]. We have observed a similar range of phenotypes in our analysis. Injection of high amounts of GSK-3 RNAi (2 µg/µL) lead to female sterility or large descrease in egg laying (data not shown), while decreasing dsRNA concentration (up to 50 ng/µL) allowed the recovery of RNAi embryos. The decrease in dsRNA concentration might affect the level of *Tc-GSK-3* knockdown in the embryos. Thus, we analyzed *Tc-GSK-3* expression in control (LacZ RNAi) and *Tc-GSK-3* RNAi eggs by RT-PCR (Figure 8A). Injection of *Tc-GSK-3* dsRNA (50 ng/µL) decreased its expression to levels of 20% of the control (Figure 8A), confirming that *Tc-GSK-3* transcription was affected. After injection of *Tc-GSK-3* dsRNA (50 ng/µL) egg laying was also reduced when compared to the control (Figure 8B), as well as the number of hatched larvae, which is largely decreased after *Tc-GSK-3* dsRNA injection (Figure 8C).

These results stimulated the analysis of the morphology of *Tc-GSK-3* RNAi embryos (Sup. Figure 1). In *T. castaneum* control embryos, the first visible differentiation event can be visualized by nuclear DAPI stainings. At that stage smaller nuclei at the posterior-ventral region correspond to the future embryo and the amnion, while the large nuclei of the serosa are detected at the anterior-dorsal region [49], [50]. In contrast, *Tc-GSK-3* RNAi embryos fail to develop a proper serosa and show an expansion of the germ rudiment and a smaller anterior serosa, as previously noticed by [44]. We also noticed that about 20% of the embryos stop development before cellularization, suggesting an essential role of *Tc-GSK-3* during early embryogenesis (data not shown), when glycogen levels are high. Later in development, between 40–44 hours, control embryos show clear leg buds (arrows) and segmental grooves (arrowheads) (Sup. Figure 1A). In contrast, *Tc-GSK-3* RNAi embryos lack both features, suggesting that the segmentation cascade is affected (Sup. Figure 1B). In addition, ventral midline appears affected at least in some RNAi embryos (Sup. Figure 1C -arrow), suggesting a possible role of *Tc-GSK-3* in midline patterning.

All the aforementioned results can be explained by a morphogenetic role of *Tc-GSK-3* as a downstream of the Wnt pathway. We investigated if knockdown of *Tc-GSK-3* would lead to changes in the metabolic status of *T. castaneum* embryos, particularly in glycogen content. Interestingly, *Tc-GSK-3* RNAi embryos show higher glycogen level than the control (LacZ RNAi) embryos (Figure 8D). Since oviposition is affected after *Tc-GSK-3* RNAi (Figure 8B), we normalized glycogen content of each sample to protein levels, which does not extensively change in the period analyzed (Sup. Figure 2 - 48–72 hours). This result suggests that besides its morphogenetic role, *Tc-GSK-3* also plays a role during embryonic metabolism.

Altogether our results define at least two important conclusions about metabolic control during *T. castaneum* early embryogenesis. First, the mother deposit high levels of glycogen in the egg, which is largely used up during the first eight hours of development when cleavages and cellularization takes place. Two key enzymes involved in glucose and glycogen metabolism *Tc-Hex* and *Tc-GSK-3,* respectively, are maternally provided as mRNA and seem to be essential for early embryogenesis. A second period of embryonic development occurs after 24 hours of embryonic development when glycogen levels decrease and glucose increase. At that stage since *Tc-GSK-3* and *Tc-Hex* expression is confined to the embryonic tissue, we suggest that different regions of the egg display different metabolic activities.

## Discussion

### Glucose and Glycogen Content are Tightly Regulated during *T. castaneum* Embryogenesis

Analysis of glucose (Figure 2) and glycogen content (Figure 6) during *Tribolium* embryogenesis suggests that both storage molecules are supplied maternally and are consumed during early cleavages and blastoderm formation. Previous analysis in ticks also showed high glycogen levels and low glucose content at early stages of embryogenesis. In ticks, glucose and glycogen seems to be upregulated after blastoderm formation and germ band elongation [8], suggesting that gluconeogenesis occurs during tick embryogenesis. In mosquitos during early embryogenesis glucose is converted by Hex and, most likely, driven to the pentose-phosphate pathway (PPP), which will generate the nucleotides required for the intense nuclei division during syncytial blastodermal stage. It is possible that a similar regulation occurs in *T. castaneum* since glucose is also slightly reduced during the first 8 hours of embryogenesis and increases only after 20 hours of development (Figure 2). Glycogen regulation appears to be different between *T. castaneum* and the other insects which are blood feeders. In *T. castaneum* glycogen does not accumulate, but is further downregulated during the late phase of embryogenesis. *T. castaneum* larvae hatch with low glycogen levels (Figure 6), which might be related to the fact that starch is readily available after hatching in this beetle. In contrast, mosquito and tick larvae might remain without feeding for a long period, thus, requiring an efficient glycogen storage system after hatching.

### Tissue Specific-expression of *GSK-3* and *HexA1* as a Possible Explanation for Antagonistic Metabolic Reactions during Embryogenesis

Genes responsible for metabolic reactions are generally not regulated at the level of transcription, they are considered to be housekeeping genes constitutively transcribed throughout life (*e.g.* [51]). *In situ* hybridization expression analysis of *Tc-GSK-3* (Figure 7) and *Tc-HexA1* (Figure 4) shows that these genes display spatial and temporal regulation. Indeed, after gastrulation, 8–12 hours onwards, both genes are specifically expressed at the embryonic tissue and not in the extraembryonic cells (Figure 4E and 7C,D). These results suggest that metabolism of the embryonic cells might be different from the extra-embryonic cells. One of the basic differences among these two cell populations is that the serosa cells do not undergo cell proliferation and cytokinesis but rather become polyploid and stop cell division [49]. In contrast, embryonic cells are highly proliferative and express *Hexokinase* (*Tc-HexA1*) mRNA as judged by *in situ* hybridization (Figure 4A,B). Hexokinase activity is high at the first four hours of embryogenesis when the fast cleavages take place (Figure 4F – 0–4 hours).

Proliferating cells including embryonic tissues are thought to rely on aerobic glycolysis, or on the metabolism of glucose to lactate under oxygenated conditions, to assist in the synthesis of biosynthetic precursors necessary for growth and embryonic/progenitor like state [52]. For instance, human pluripotent stem cells maintain high glycolytic rates with high levels of hexokinase II and inactive pyruvate dehydrogenase [53].Thus, it is possible that the high Hex activity we observe during the first hours of *T. castaneum* embryonic development drives G6P to several biochemical pathways including the pentose pathway or aerobic glycolysis like it has been described for tumor cells. Thus the large decrease in glycogen content at early hours (Figure 6) might be driven to the pentose pathway or aerobic glycolysis without noticeable changes in glucose levels (Figure 2).

Interestingly, variations in Hex activity during embryogenesis as we observed here (Figure 4F) have also been reported in the frog *Xenopus laevis* [54]. In *Xenopus* Hex activity could be rate-limiting at relatively late developmental stage before hatching. In conclusion the regulation of *Tc-HexA1* and *Tc-GSK-3* expression pattern reported here might be important for the overall metabolic status during embryogenesis.

### *Hexokinase* Duplication and Evolution

Our results also highlight an important feature of the *Hex* locus in *T. castaneum. Hexokinase* locus displays two paralogs (*Tc-HexA1* and *Tc-HexA2*) located in tandem in the beetle genome (Figure 3A). Interestingly, both genes appear to be expressed during embryogenesis (Figure 3A), although *Tc-HexA1*seems to be the only one activated during early stages of development. *Hex* duplication has also been reported in *Drosophila melanogaster*, where four *Hex* genes are present in the genome. Interestingly, flight muscle Hexokinase-A (Hex-A) is the most conserved and essential hexokinase isozyme among *Drosophila* species [24], [26]. The other three hexokinases in *D. melanogaster* are expressed in the fat body (HexC) and in the testis (Hex-t1 and Hex-t2) suggesting that *Hex* duplication and putative metabolic changes might be more frequent than previously thought.

Our results confirm that *HexA* is the ancestral *Hex* in insects (Figure 3B). Tc-HexA1 and Tc-HexA2 are highly similar and most amino acid changes can be observed in non-structured regions *i.e.* in residues not important for the interaction with Glucose or Glucose-6-P, suggesting that both enzymes might perform similar functions in *T. castaneum* (data not shown). Although we did not analyze the expression of *Tc-HexA2* in detail, it is interesting to note that Hex activity is high at 24 and 30 hours (Figure 4F), when the transcription of *Tc-HexA1* and *Tc-HexA2* can be observed (Figure 3A). Thus, it is possible that both genes are transcribed during the second phase of embryogenesis, after 24 hours, when segmentation has finished. Tc-HexA1 protein sequence is highly similar to Hex from other invertebrates like the shrimp *Litopenaeus vannamei.* Shrimp Hex is induced by the hypoxia inducible factor 1 (HIF-1) and displays specific tissue expression [27]. In zebrafish six hexokinases have been described which are expressed in a tissue specific manner [55]. Thus, the temporal and tissue specific expression of *Tc-HexA1* found in our study is also corroborated by findings with hexokinases in other model systems.

### *Tc-GSK-3* Expression and Function are Correlated to Changes in Glycogen Levels during Embryogenesis

**Figure 9:**
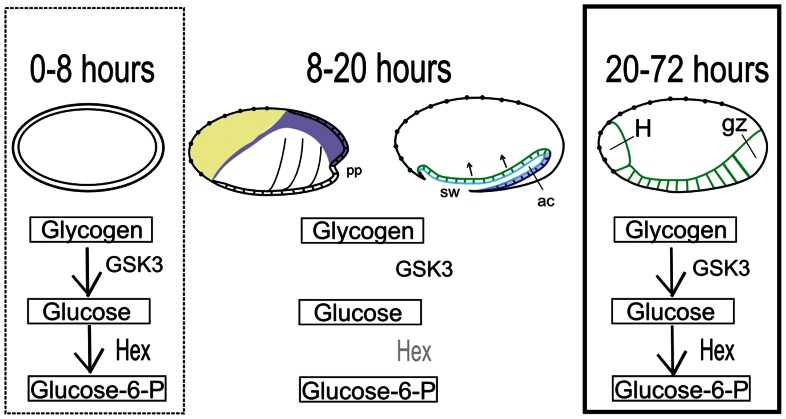
A simplified model for the regulation of glucose and glycogen during early *T. castaneum* embryonic development. Three distinct time points of *Tribolium castaneum* development are highlighted (0–8, 8–20 and 20–72 hours). During the first eight hours of development fast cleavages occur and glycogen content maternally provided is degraded. Hexokinase activity and *Tc-HexA1* mRNA expression is high. Between 8–16 hours of development the extra-embryonic membranes, amnion (purple) and serosa (yellow) are established and *Tc-HexA1* and *Tc-GSK-3* expression are restricted to the embryonic tissue (see text for details). During these stages (8–20 hours) glycogen and glucose levels remain largely stable. Between 20–72 hours glycogen is degraded and glucose levels increase, as well as Hex activity. At 20 hours AEL head (H) and the posterior growth-zone (gz) can be visualized. pp – posterior pit, sw – serosal window, ac- amniotic cavity. For a morphological description of the embryonic events see [49].

Besides its role in glycogen synthase regulation, *GSK-3* is also an important downstream component of the Wnt pathway [56]. Recently, *Tc-GSK-3* was shown to be essential for early AP patterning during *T. castaneum* embryogenesis; *Tc-axin*, an inhibitor of the Wnt pathway, is maternally provided and localized at the anterior [44]. Thus, canonical Wnt signaling must be carefully regulated along the AP axis in *T. castaneum* in contrast to other derived insects which rely on other anterior patterning systems localized during oogenesis [57]. Here we have investigated how *Tc-GSK-3* RNAi would affect oviposition and larvae hatching; both events were largely reduced after RNAi (Figure 8A–C). Several *Tc-GSK-3* RNAi embryos stop development before cellularization (data not shown) and some display apparent defects in ventral midline (Sup. Figure 1). In addition, we have observed changes in glycogen content during embryogenesis after *Tc-GSK-3* RNAi (Figure 8D). In agreement with our results, previous studies have shown that overexpression of GSK-3 are sufficient to inactivate GS and thus decrease glycogen content in mammalian cell culture [58]. Thus, *Tc-GSK-3* appears to be an important factor which links the cell metabolic state and Wnt signaling pathway. Recently, activation of Wnt pathway and epithelial mesenchymal transition have been linked to mitochondrial repression and glycolytic switch in tumor cells [59]. Since *T. castaneum* embryos also show high activity of Hex and of the glycolytic pathway during the early proliferative stage (Figure 9), it is possible that embryonic and tumor cells display similar metabolic status.

It is important to notice that glycogen content does not vary along the whole embryogenesis but rather at two distinct decrease phases (Figure 9). In the second phase of glycogen content decrease, glucose levels increase (Figure 2), suggesting that this glucose generated might be required at larval stage. Further studies analyzing other key enzymes are required to completely dissect the regulation of the metabolic pathways during beetle embryogenesis.

### Conclusion

Our study provides the first analysis of the metabolism of the beetle *Tribolium castaneum* during embryogenesis. Hex and GSK-3 appear to be essential for oogenesis and embryogenesis as judged by our functional analysis. Importantly, Hex and GSK-3 display different mRNA expression profiles in embryonic and extra-embryonic cells suggesting that metabolic compartmentalization occurs during beetle embryogenesis.

## Supporting Information

Figure S1 **Knockdown of GSK-3 impairs** ***Tribolium*** **embryonic development.** Nuclear DAPI stainings during germ band elongation of WT (A) and of *Tc-GSK-3* RNAi embryos (B,C). (A) In Control (WT) limb buds (white arrows) and ventral midline (white arrowheads) are evident, while *Tc-GSK-3* knockdown embryos (B,C) lack limb buds and display a broader appearance. In C ventral midline appears open at later stages and segmental grooves seems absent.
(TIF)

Figure S2 **Protein concentration changes during** ***Tribolium*** **embryogenesis.** Protein content was normalized to egg number. Protein concentration is reduced during the first 4 hours of embryogenesis and increases between 20 and 24 hours. Between 24 and 48 hours a large reduction is observed. This level is maintained in the next 24 hours (48–72 hours) and not altered until hatching (data not shown).
(TIF)
