# Supplementary figures and images for: Glycogen and Glucose Metabolism Are Essential for Early Embryonic Development of the Red Flour Beetle Tribolium castaneum
Source: PLoS One. 2013 Jun 4;8(6):e65125. doi: 10.1371/journal.pone.0065125 (PMC3672164; doi:10.1371/journal.pone.0065125)

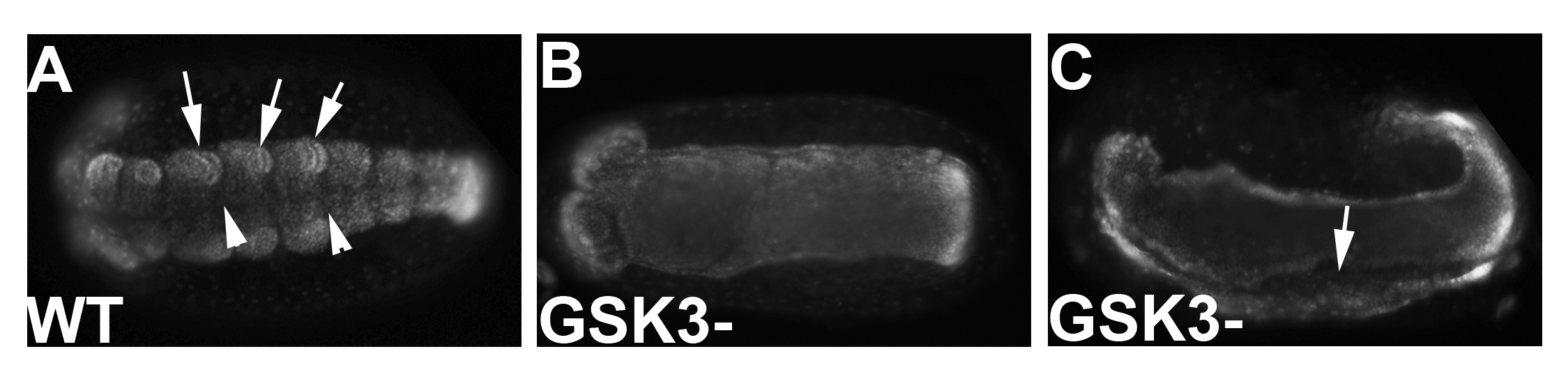

Supplement: Figure S1 — Knockdown of GSK-3 impairs Tribolium embryonic development. Nuclear DAPI stainings during germ band elongation of WT (A) and of Tc-GSK-3 RNAi embryos (B,C). (A) In Control (WT) limb buds (white arrows) and ventral midline (white arrowheads) are evident, while Tc-GSK-3 knockdown embryos (B,C) lack limb buds and display a broader appearance. In C ventral midline appears open at later stages and segmental grooves seems absent. (TIF) [file pone.0065125.s001.tif]

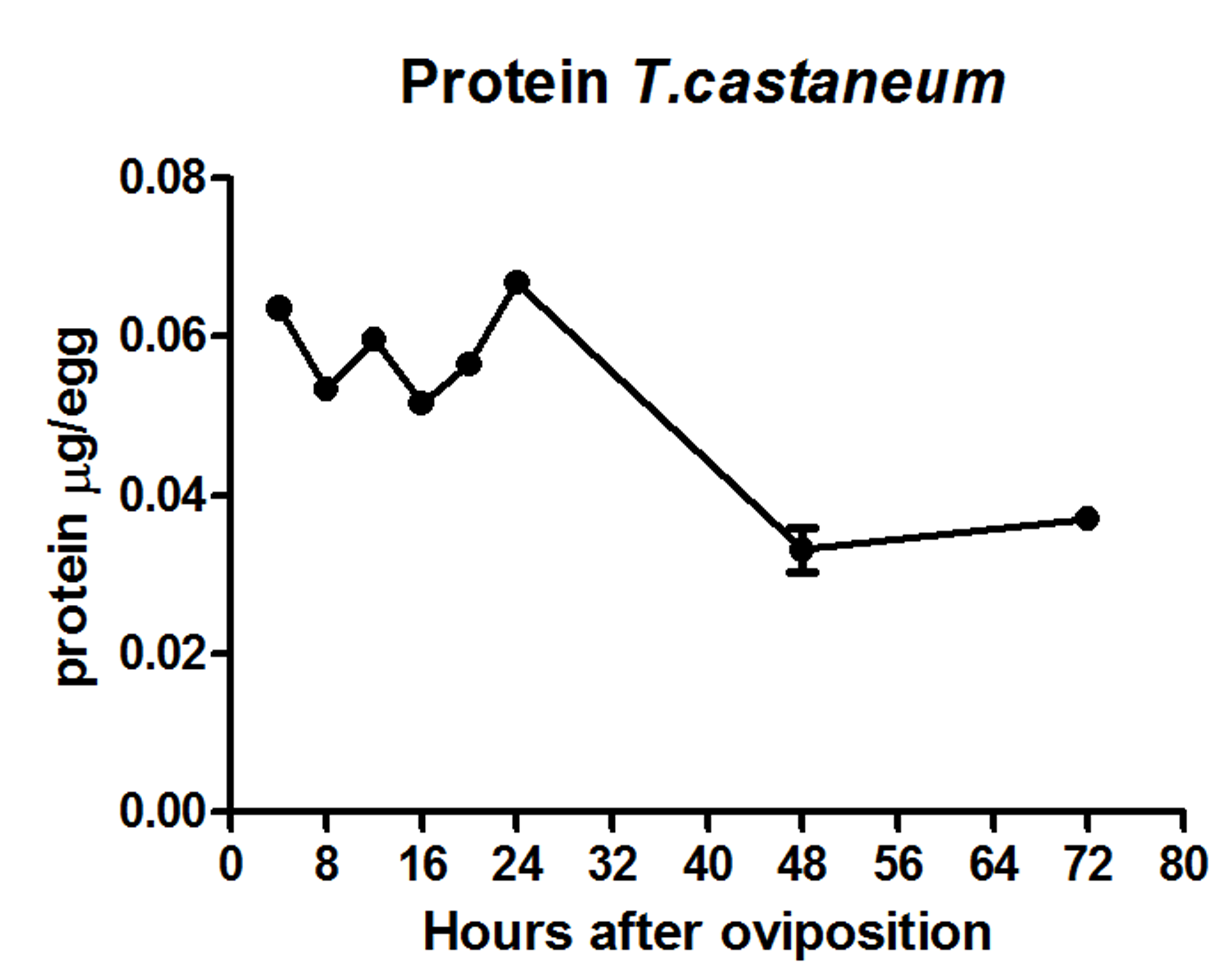

Supplement: Figure S2 — Protein concentration changes during Tribolium embryogenesis. Protein content was normalized to egg number. Protein concentration is reduced during the first 4 hours of embryogenesis and increases between 20 and 24 hours. Between 24 and 48 hours a large reduction is observed. This level is maintained in the next 24 hours (48–72 hours) and not altered until hatching (data not shown). (TIF) [file pone.0065125.s002.tif]
